# Diagnostic value of pyruvate kinase isoenzyme type M2 for colorectal cancer: Evidence from meta-analysis

**DOI:** 10.1097/MD.0000000000036942

**Published:** 2025-09-26

**Authors:** Hang Zhao, Xiaonan Sun, Ying Wang, Jing Tong

**Affiliations:** a Department of Gastroenterology, The First Affiliated Hospital of China Medical University, Shenyang, Liaoning, P.R. China; b Department of Ophthalmology, The 4th People’s Hospital of Shenyang, Shenyang, Liaoning, P.R. China.

**Keywords:** Colorectal cancer, kinase isoenzyme type M2, meta-analysis, sensitivity, specificity

## Abstract

**Background:**

This meta-analysis is to objectively and comprehensively evaluate the diagnostic value of pyruvate kinase isoenzyme type M2 (M2-PK) in colorectal cancer (CRC).

**Methods:**

All studies on the diagnostic value of M2-PK for CRC were retrieved from the PubMed, WanFang, CNKI, MEDLINE, Web of Science, and CBM databases until February 2020. Data were extracted from studies conforming to the inclusion and exclusion criteria. The sensitivity and specificity data were pooled. For heterogeneity analysis, subgroup analysis was performed according to race, test method, sample type, and tumor staging. The Stata 16.0 was used to conduct the statistical analysis.

**Results:**

About 27 studies were included: 1821 cases in the case group and 3406 cases in the control group. The results showed that the pooled sensitivity and specificity of M2-PK for diagnosing CRC were 77% (95% CI: 73%–81%) and 83% (95% CI: 78%–87%), and the area under the ROC curve was 87% (95%CI: 83%–89%).

**Conclusion:**

This meta-analysis showed that M2-PK has a relatively high sensitivity and specificity for diagnosing CRC and has a higher diagnostic value in early diagnosis of CRC. However high-quality prospective studies are required to confirm its diagnostic value.

## 1. Introduction

Colorectal cancer (CRC) is one of the most common malignant tumors of the digestive system. Early CRC has a good treatment effect, while patients with advanced CRC have a poor treatment effect and a low survival rate. At present, there is a lack of a superior and more convenient tumor marker for the early detection, early diagnosis, and monitoring of treatment progression in CRC.

Colonoscopy is currently the gold standard for early diagnosis of CRC, but as an invasive test, patient compliance is low. In addition, there are also some important methods for CRC screening, including gFOBT, iFOBT, pyruvate kinase isoenzyme type M2 (M2-PK), carcinoembryonic antigen (CEA), carbohydrate antigen 199 (CA199), carbohydrate antigen 724 (CA724), etc., but each of them has certain limitations. The current research does not have a clear choice of methods, so further analysis of these methods is needed to determine an optimal method to fill the gaps in the field.

M2-PK, as an isoenzyme of pyruvate kinase, mainly exists by the form of tetramer in normal cells. However, due to the needs of anabolic metabolism, M2-PK is overexpressed and exists mainly as a dimer in tumor cells of almost different tissue origin. The tetramer form M2-PK has a high affinity for the substrate phosphoenol-pyruvate (PEP), while the dimer form has a low affinity for the substrate PEP. The dimerization group induced by glycolysis phosphoric acid metabolite fructose 1, 6-diphosphate (FBP) was tetramer.^[1]^ Some studies reported that M2-PK in stool and plasma can be a tool for early diagnosis of CRC.^[2–5]^ However the clinical diagnosis value of M2-PK was not completely consistent with each study in sensitivity and specificity. Therefore, we made a meta-analysis from published trials to investigate the value of tumor M2-PK as a biomarker for the diagnosis of CRC. The study would provide valuable reference for clinical research and application of M2-PK in CRC.

## 2. Methods

### 2.1. Literature search and inclusion criteria

A reasonable literature search of PubMed, WanFang, CNKI, MEDLINE, Web of Science, and CBM databases was carried out for articles. The search terms used included Pyruvate Kinase, Pyruvate Kinase M2, M2-PK, colorectal cancer, colon cancer, and rectal cancer. All full-texts had been screened, and that could guarantee the eligibility of all articles to involve our study.

Studies that met the following criteria: formally published and written in English or Chinese until February 2020; CRC was confirmed by colonoscopy and/or histology, and the articles included the sensitivity and specificity results of M2-PK in our study.

Studies were excluded due to the following reasons: article type as reviews, letters, case reports, conference data, and editorials; articles had insufficient data, and duplication data.

### 2.2. Data extraction and quality assessment

Two professional reviewers conducted data extraction from texts, tables, and figures independently. They also completed the quality assessment of the research articles. Any disagreement was resolved through discussion with others. The baseline characteristics were collected: first author, published year, country, index test, specimen, cutoff value, cases of number, sensitivity, and specificity.

The authors used Stata software version 16.0 to assess the quality of included studies and made the forest plot of meta-regression and subgroup analyses.

### 2.3. Statistical analyses

All the analyses were undertaken by Stata software version 16.0 and SPSS Statistics 21. As a diagnostic meta-analysis, the evaluation indicators are pooled sensitivity, specificity, positive likelihood ratio (+LR), negative likelihood ratio (−LR), and calculate the area under the ROC curve (AUC). Simultaneously we can pooled the receiver operating characteristic curve (ROC) and AUC. The heterogeneity of the effect was evaluated by the Chi-square. A Deek quantitative funnel plot asymmetry test was used to determine and evaluate whether there was publication bias in this research.

## 3. Results

### 3.1. Description of studies

**Figure 1.**
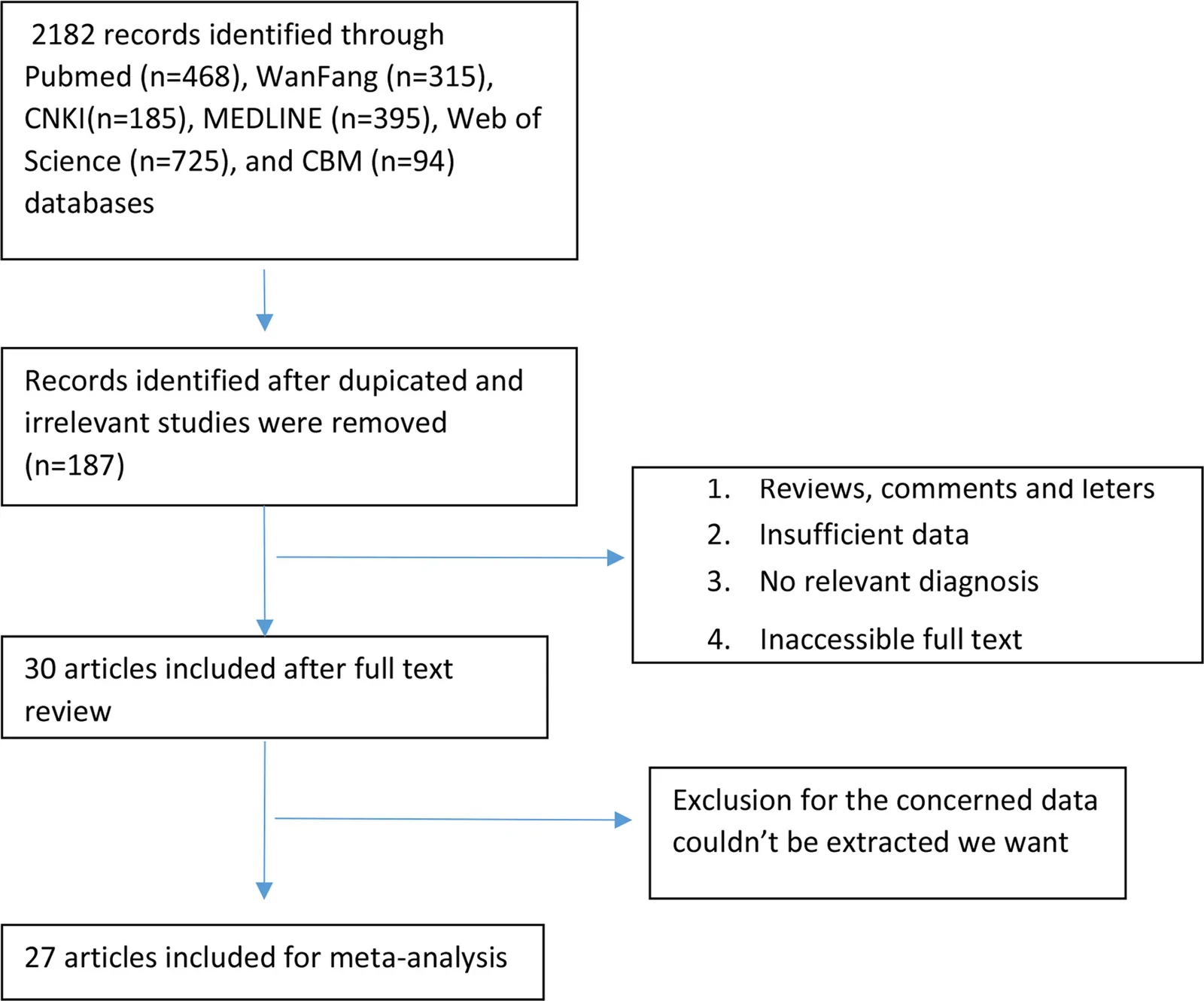
Study flow diagram for this meta-analysis.

A total of 2182 articles on M2-PK diagnosis and CRC were obtained in the preliminary examination. Among those, there were 468 from PubMed, 315 from WanFang, 185 from CNKI, 395 from MEDLINE, 725 from Web of Science, and 94 from CBM. Based on inclusion and exclusion criteria, 187 articles concerning the M2-PK and CRC were primarily identified from 6 databases (Fig. 1). After duplicated and irrelevant articles were excluded, we screened the articles selected for full-text review for the following reasons: reviews, letter, or irrelevant research topic, without valuable data of nonfull test. Finally, we selected the following 27 articles as our final research objects (Table 1).

**Table 1:** The main characteristics of the 27 studies included in the meta-analysis.

ID	References	Country	Index test	Cutoff (U/mL)	Specimen	Case number	Se%	Sp%
1	Loktionov et al^[6]^	English	ELISA	8.97	Colorectal mucus	18	76.50	94.30
2	Yu et al^[7]^	China	Colloidal gold method	–	Stool	39	64.10	85.30
3	Dabbous et al^[8]^	Egypt	ELISA	4	Stool	20	85.00	75.00
4	Ni and Xiaolin^[9]^	China	ELISA	15	Plasma	137	66.42	88.27
5	Longfei et al^[10]^	China	ELISA	25.2	Plasma	231	62.30	81.50
6	Gang and Lijuan^[11]^	China	ELISA	15	Plasma	80	73.80	86.10
7	Rutka et al^[12]^	Hungary	Immunochromatographic	4	Stool	19	94.70	67.50
8	Fung et al^[13]^	Australia	ELISA	–	Plasma	98	73.00	95.00
9	Sithambaram et al^[14]^	Malay Chinese Indian	ELISA	–	Plasma	100	93.00	97.50
10	Kim et al^[15]^	Korea	Immunochromatographic	4	Stool	139	92.80	83.30
11	WeiJuan et al^[16]^	China	ELISA	15	Plasma	83	57.83	77.78
12	Wei^[17]^	China	ELISA	10	Stool	74	71.60	60.00
13	Abdullah et al^[18]^	Javanese Malay	ELISA	4	Stool	42	71.40	71.00
14	Parente et al^[19]^	Italian	ELISA	4	Stool	47	87.20	62.60
15	Meng et al^[20]^	China	ELISA	4	Plasma	93	81.72	74.05
16	Yu et al^[21]^	China	ELISA	4	Stool	33	78.8	80.7
17	Yong and Junjiang^[22]^	China	ELISA	4	Stool	44	63.60	81.80
18	Shastri et al^[23]^	Germany	ELISA	4	Stool	55	78.20	73.80
19	Lianzhou et al^[24]^	China	ELISA	4	Stool	56	75.00	94.10
20	Haug et al^[25]^	Germany	ELISA	4	Stool	65	68.00	79.00
21	Koss et al^[26]^	English	ELISA	3.33	Stool	32	91.00	92.00
22	Mulder et al^[27]^	The Netherlands	ELISA	4	Stool	52	85.00	90.00
23	Zhang et al^[28]^	China	ELISA	10	Stool	80	77.50	92.50
24	Guan-Fu et al^[29]^	China	ELISA	4	Stool	43	65.10	88.40
25	Tonus et al^[30]^	Germany	ELISA	4	Stool	54	78.00	93.00
26	Hardt et al^[31]^	Germany	ELISA	4	Stool	60	73.00	78.00
27	Naumann et al^[32]^	Germany	ELISA	4	Stool	27	85.20	65.00

ELISA = enzyme-linked immunosorbent assay, Se = sensitivity, Sp = specificity.

The 27 articles contained 1821 patients with CRC and 3406 healthy controls. The gold standard for all patients diagnosed with CRC was histopathology after colonoscopy or surgery. The patient’s colorectal mucosa, stool, and plasma as samples were detected M2-PK expression. The characteristics of the data were collected in the research (Table 1).

### 3.2. Literature quality assessment

**Figure 2.**
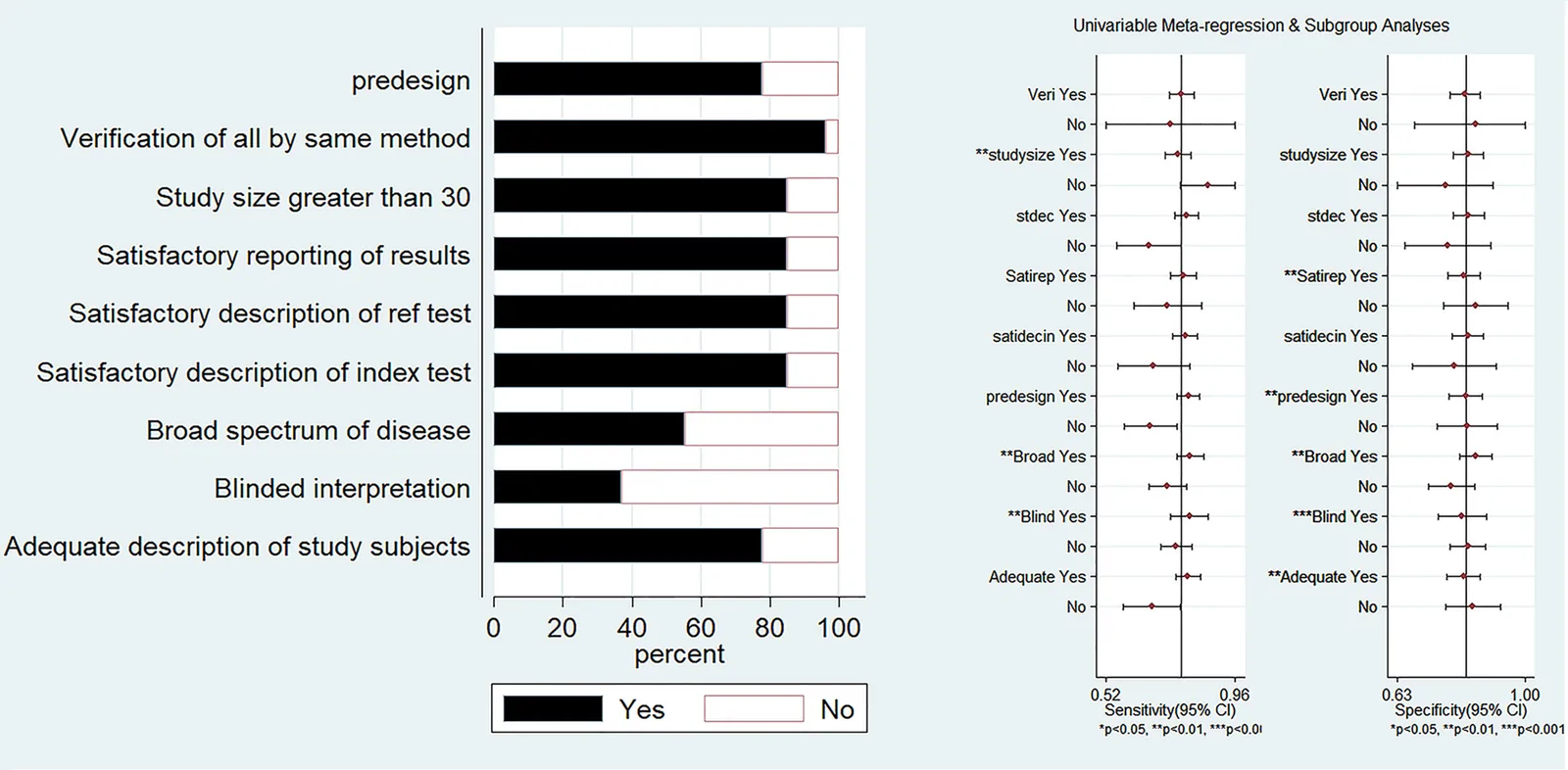
Quality evaluation chart for all the included studies and the forest plot of Meta-regression & subgroup analyses.

All 27 studies were completed the quality assessment through the following aspects: predesign, verification of all by same method, study size >30, satisfactory reporting of results, satisfactory description of ref test, satisfactory description of index test, broad spectrum of disease, blinded interpretation, and adequate description of study subjects (Fig. 2).

### 3.3. The study of meta-analysis

**Figure 3.**
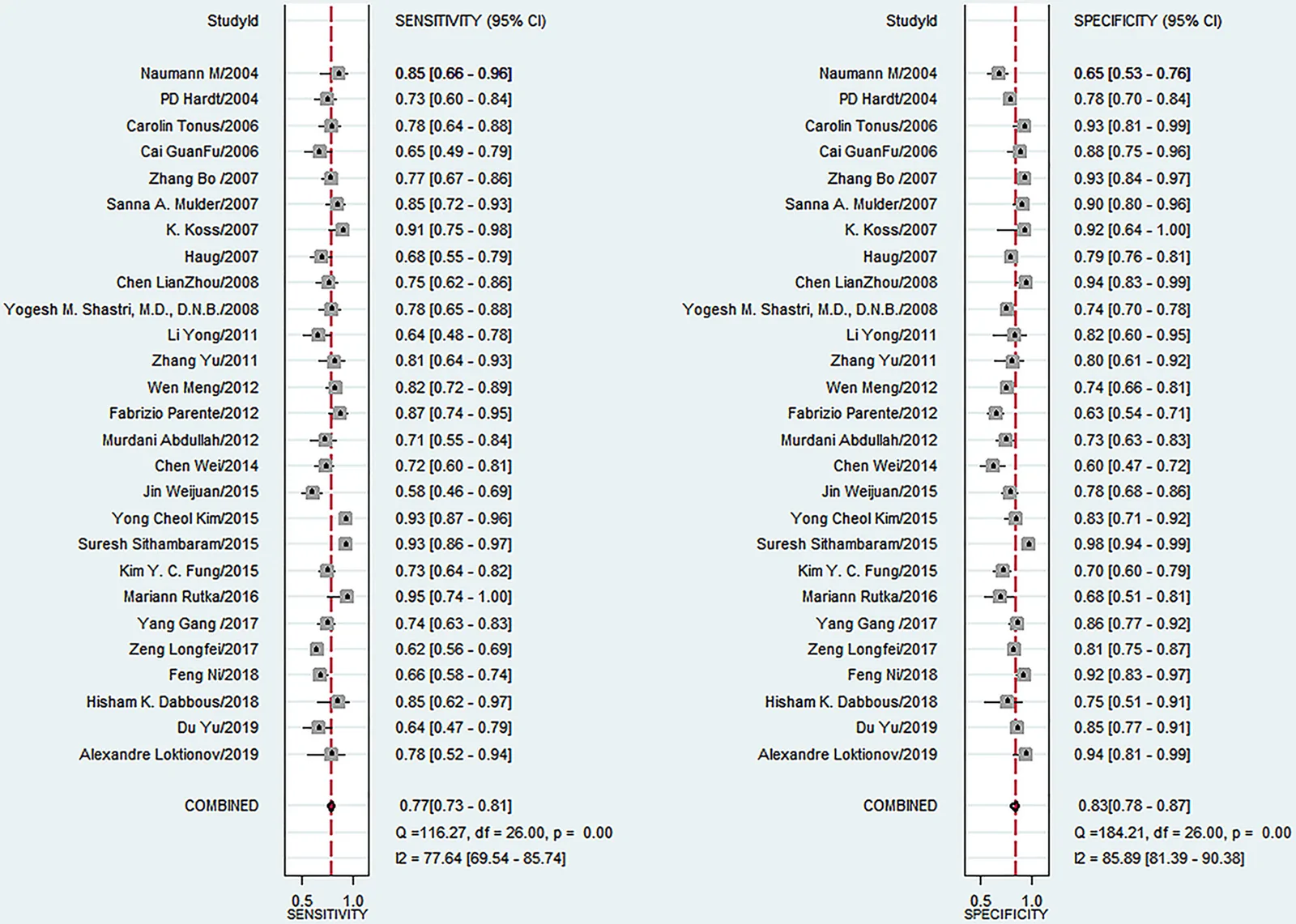
Forest plot showing the sensitivity and specificity for individual studies with their 95% confidence intervals, pooled sensitivity, and pooled specificity from all the included studies.

**Figure 4.**
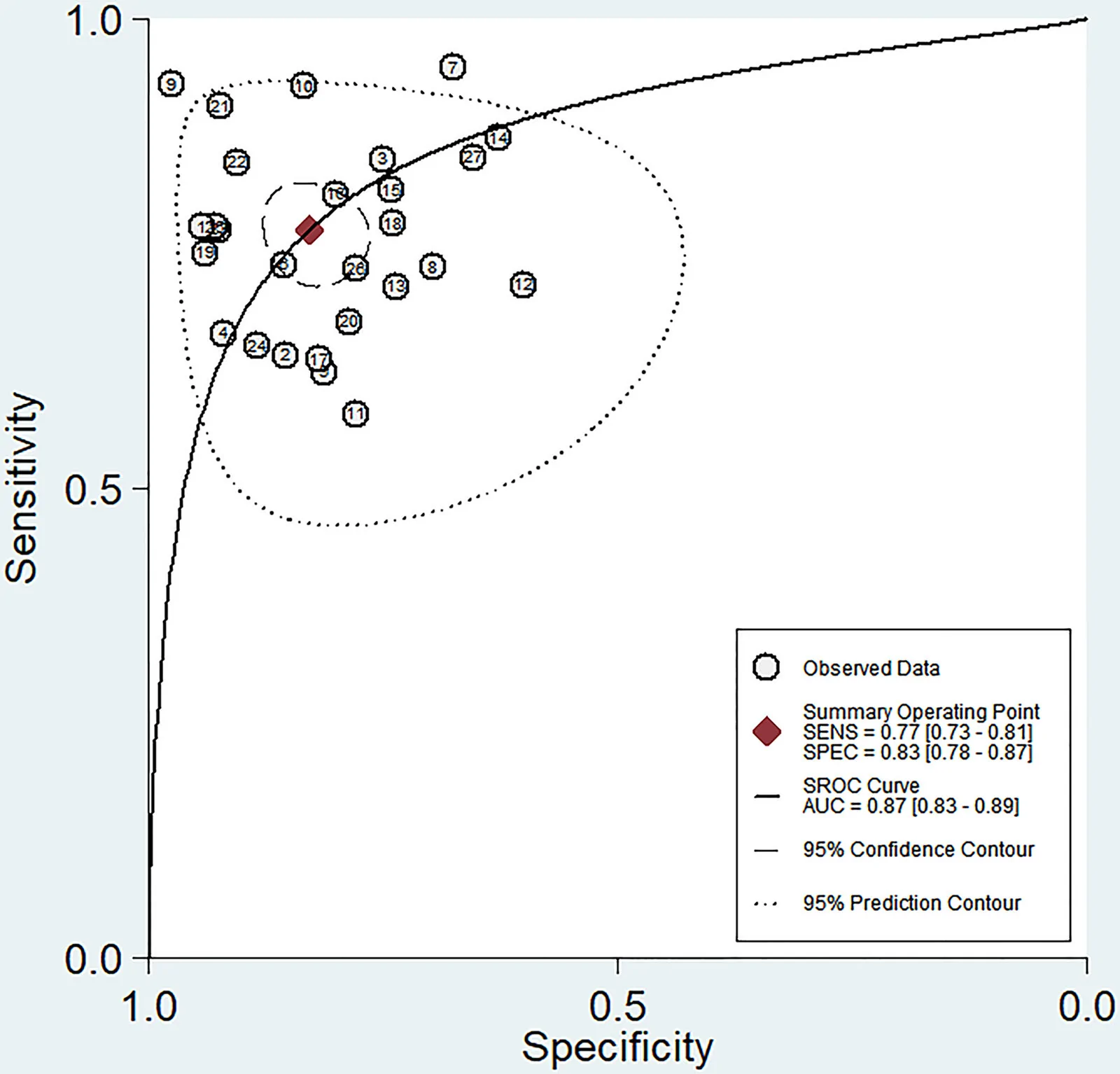
SROC curve of M2-PK assay for all the included studies.

**Figure 5.**
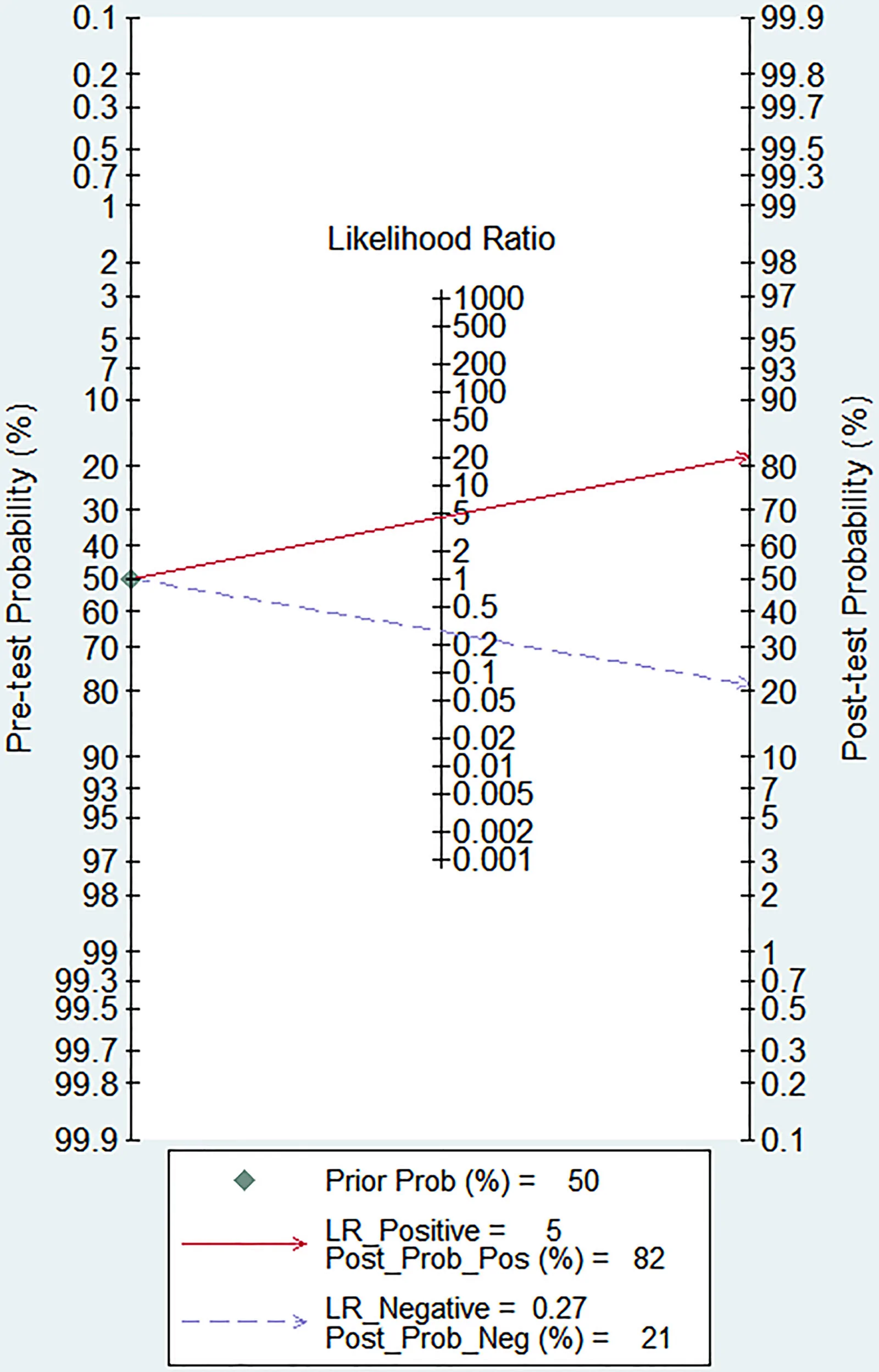
Fagan nomogram in assessment of the test probabilities for M2-PK assay.

Pooling of data demonstrated the pooled sensitivity of 77% (95% CI: 73%–81%, *P* = .00), the pooled specificity of 83% (95% CI: 78%–87%, *P* = .00) (Fig. 3), and AUC of 87% (95% CI: 83%–89%) (Fig. 4). The results suggested that M2-PK assay has relatively high value in clinical diagnosis of CRC. We got that the posttest probability was 82% by making the Fagan nomogram. pretest and posttest probability calculations illustrated that M2-PK assay can improve the diagnosis rate of CRC (Fig. 5).

### 3.4. Heterogeneity test and subgroup analysis

**Figure 6.**
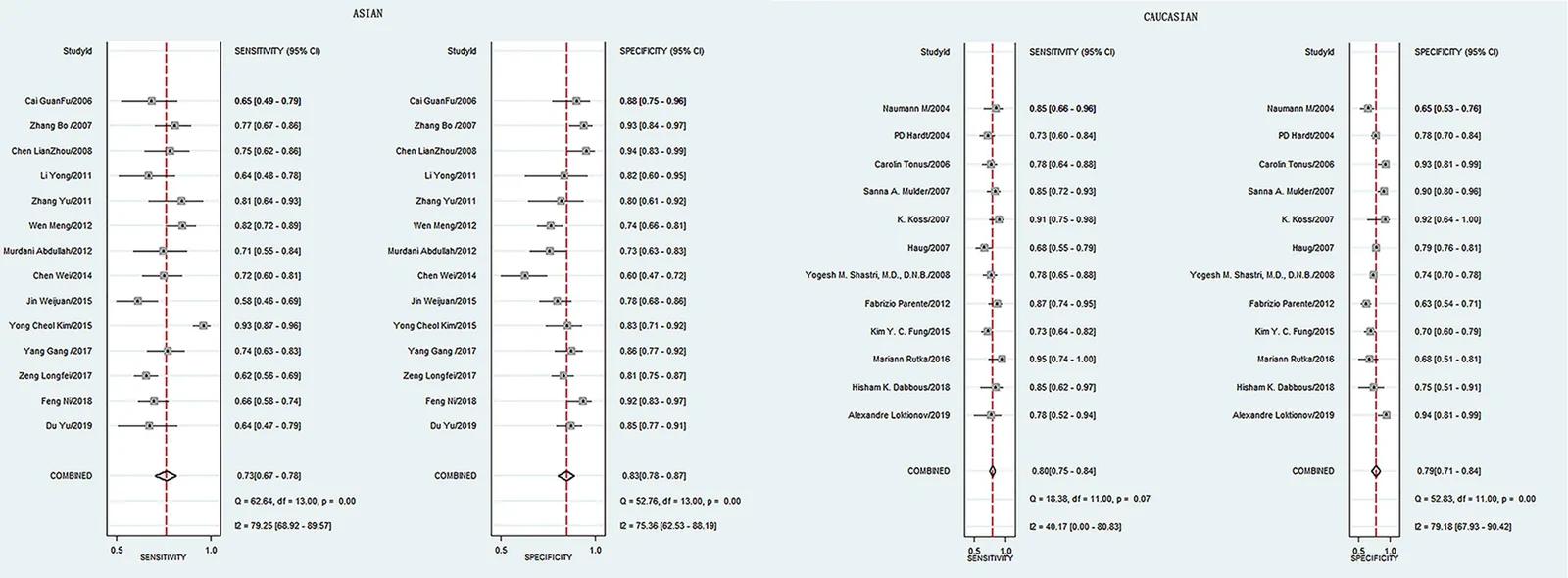
Forest plot showing the sensitivity and specificity for individual studies with their 95% confidence intervals, pooled sensitivity, and pooled specificity respectively from Asian and Caucasian.

**Figure 7.**
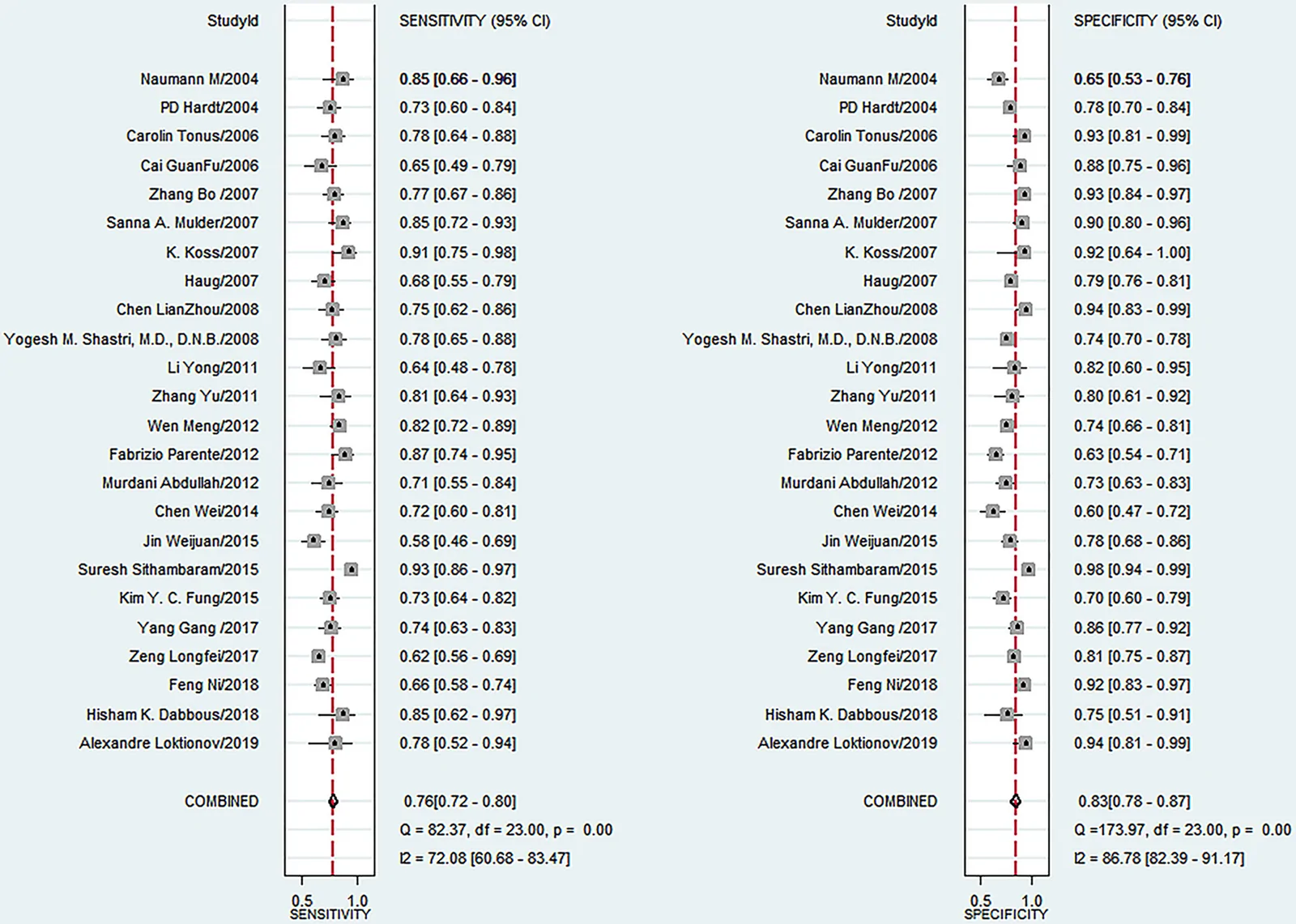
Forest plot showing the sensitivity and specificity for individual studies that M2-PK assay using enzyme-linked immunosorbent assay (ELISA), pooled sensitivity, and pooled specificity.

**Figure 8.**
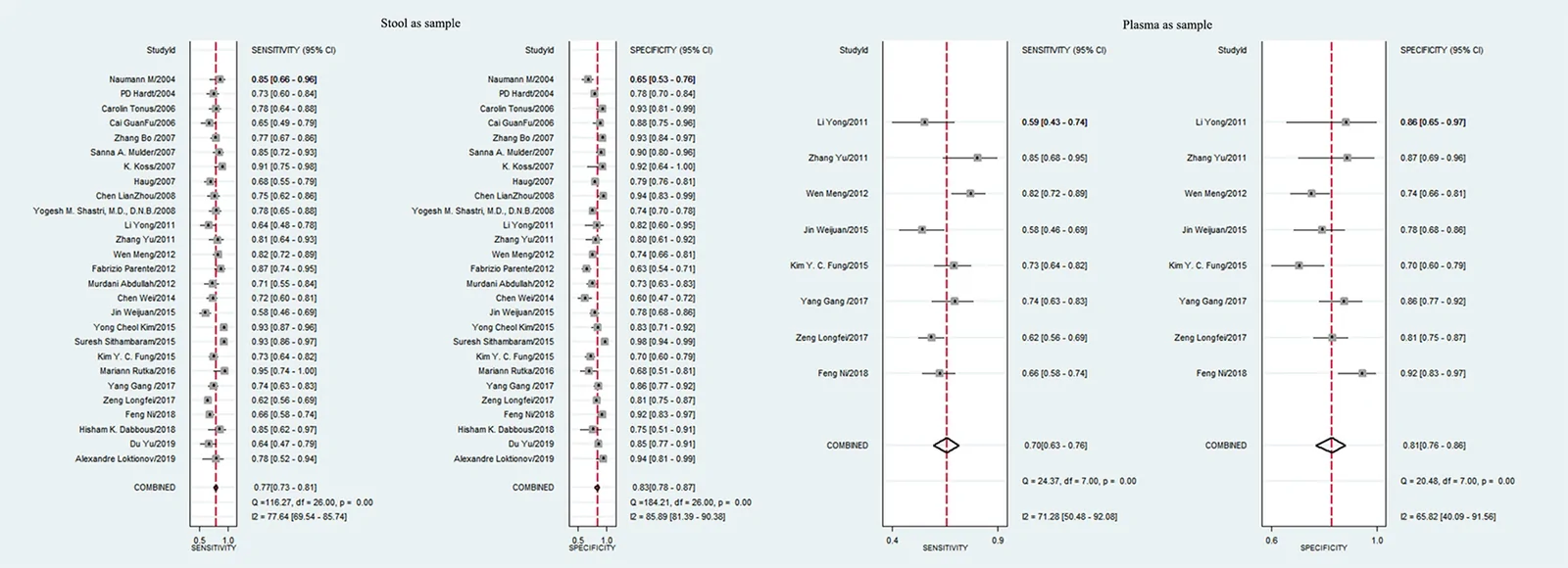
Forest plot showing the sensitivity and specificity for individual studies, pooled sensitivity, and pooled specificity that respectively used stool and plasma as samples.

**Figure 9.**
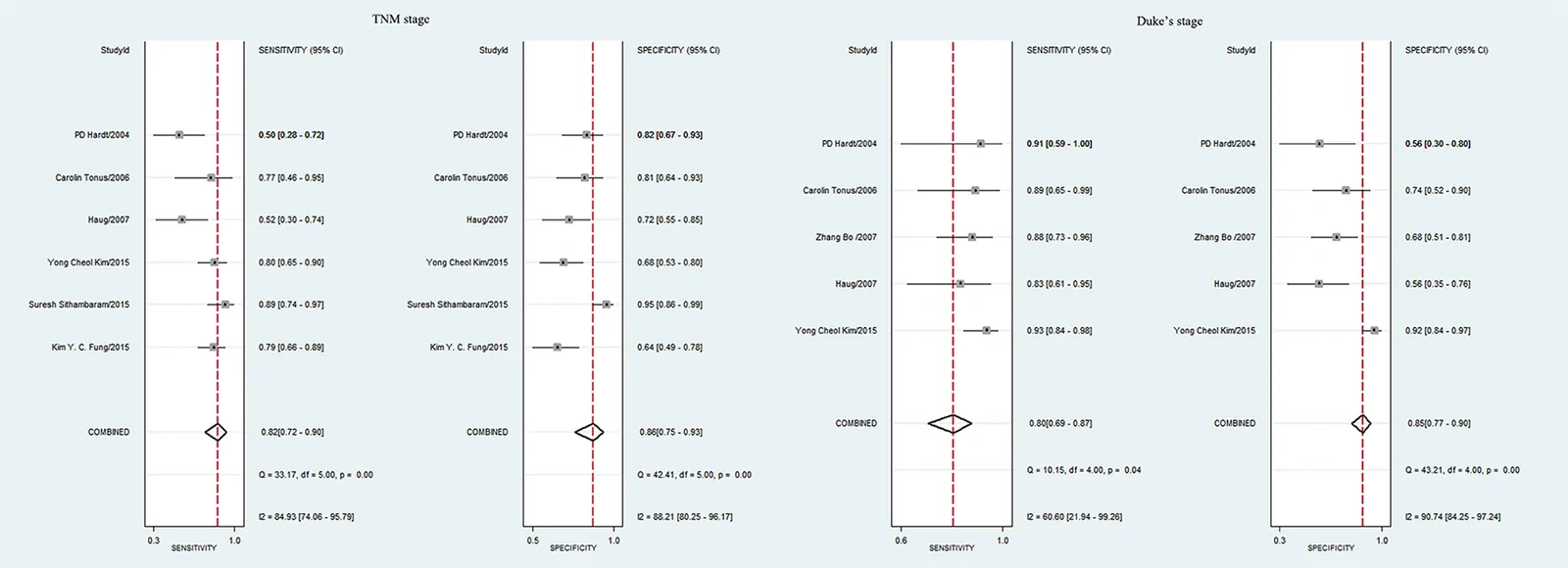
Forest plot showing the sensitivity and specificity for M2-PK assay in different TNM stages and Duke stages, pooled sensitivity, and pooled specificity.

The pooled sensitivity with *I*^2^ = 77.64 (68.54–85.74), *P* = .00, and the pooled specificity with *I*^2^ = 85.89 (81.39–90.38), *P* = .00 demonstrated that there was considerable heterogeneity in the included studies (Fig. 3). SROC curve we made was not “shoulder-arm-shaped” (Fig. 4), which could indicate that the heterogeneity among included studies were caused by nonthreshold effects. For further explore the source of heterogeneity, subgroup analysis was performed according to race (Fig. 6), test method (Fig. 7), sample type (Fig. 8), and tumor staging (Fig. 9). The heterogeneity in Caucasian was significantly decreased (*I*^2^ = 40.17, *P* = .07), but significant heterogeneity still existed in Asian (*I*^2^ = 79.25, *P* = .00). The heterogeneity in Duke stage was significantly decreased (*I*^2^ = 60.60, *P* = .04), but significant heterogeneity still existed in TNM stage (*I*^2^ = 84.93, *P* = .00). And there was no significant improvement in heterogeneity of included studies in other subgroups.

### 3.5. Publication bias

**Figure 10.**
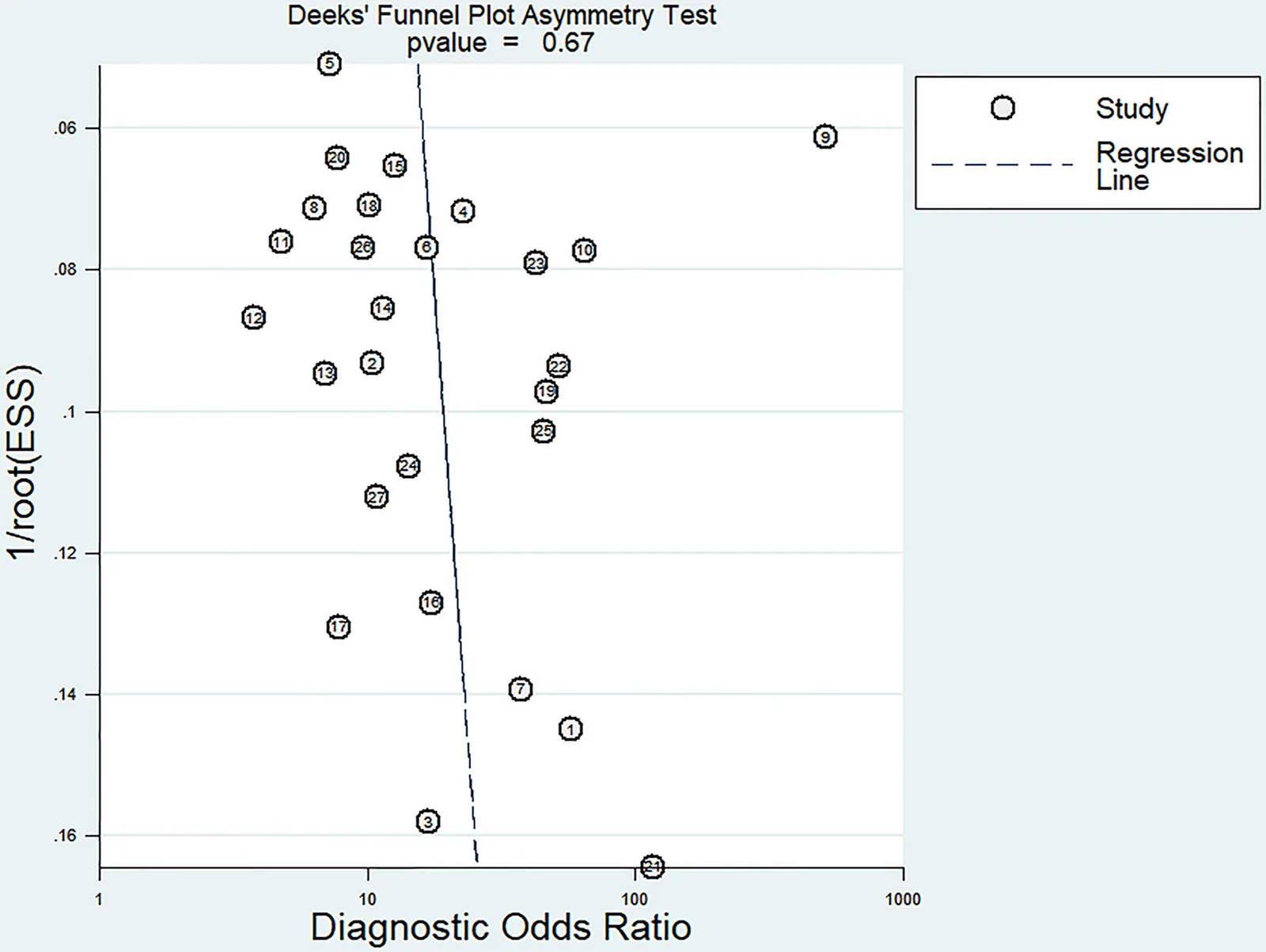
Deek funnel plot to evaluate publication bias of included studies.

Deek funnel plot asymmetry test was performed to evaluate publication bias (Fig. 10). We obtained the test *P* value = 0.67, which suggesting that there was no publication bias among the studies.

## 4. Discussion

CRC is a common malignant tumor that seriously harms human health. Early diagnosis and treatment are great significance for improving the treatment effect and the prognosis. The expression level of tumor markers will change during the development of tumors. Detecting their levels is helpful for the diagnosis, classification, treatment, and prognosis of tumors. Convenient and effective diagnostic methods have been increasingly used in clinical diagnosis of tumors.^[33]^ M2-PK has 2 forms of dimer and tetramer. Lowly active dimers are mainly involved in the synthesis of carbohydrates. Highly active tetramers are mainly involved in energy metabolism of anaerobic glycolysis. M2-PK is highly expressed in tumors and exists as a dimer in tumors with high specificity, therefore M2-PK is also called TuM2-PK. TuM2-PK is produced by tumor cells itself, and TuM2-PK is released into the blood as CRC cells proliferate and metastasize, and its level in plasma is significantly increased.^[34,35]^

All 27 studies on the application of M2-PK assay in the diagnosis of CRC were included in the research. The results showed a pooled sensitivity and specificity of M2-PK for diagnosing CRC were 77% and 83% respectively, and the AUC of SROC curves was well for diagnostic standard of CRC at 0.87. Regarding the other traditional plasma markers, the pooled sensitivity and specificity of plasma CEA were 71% and 88% respectively, in other 23 independent studies with a cutoff value of 5 µg/L.^[36]^ However, the sensitivity and specificity of CA199 were 47.8% and 69.1%, respectively.^[37]^ The sensitivity of M2-PK was significantly higher than CEA (*P* = .04), and there was no significant difference between the specificity of M2-PK and CEA. The accuracy of the M2-PK assay also appeared to be significantly stronger than CA199. That suggesting M2-PK assay has relatively higher diagnostic accuracy for CRC with moderate diagnostic performance.

Our study fills in the gaps in plasma samples from previous researchers who studied stool samples in their meta-analysis. In grouped analyses stratified by plasma and stool, the pooled sensitivity and specificity of the stool M2-PK were 0.77 (95% CI, 0.73–0.81) and 0.83 (95% CI, 0.78–0.87), and those of plasma M2-PK were 0.70 (95% CI, 0.63–0.76) and 0.81 (95% CI, 0.76–0.86). By the sensitivity and specificity of stool M2-PK comparing with those of plasma M2-PK, no statistical significance was reached (*P* = .104 and 0.859, respectively). Although plasma M2-PK has been reported to be elevated in CRC patients, it is not a specific marker. It is also higher in other types of cancer, including breast, lung, ovarian, and thyroid cancers.^[18,38,39]^ And the sensitivity of plasma M2-PK is lower than stool M2-PK. Our results for plasma M2-PK are supported by these studies. That suggesting stool M2-PK is more convenient than plasma M2-PK for diagnosis of CRC.

Hardt PD had confirmed the amount of fecal tumor M2-PK has a strong correlation with tumor stage.^[31]^ Our meta-analysis showed sensitivity and specificity of M2-PK were closely relative with TNM stage and Duke stage of CRC. If the stage of CRC was higher, its sensitivity and specificity were higher (Fig. 9).

Heterogeneity is an inevitable problem in all meta-analysis studies.^[40]^ We found some heterogeneity in the sensitivity and specificity of M2-PK assay among the included studies (Fig. 3). By the Deek funnel plot asymmetry test the effect of publication bias was conducted, but it did not indicate publication bias (*P* = .67). As shown in the research, SROC curve we made was not “shoulder-arm-shaped” (Fig. 4), which could indicate that the heterogeneity due to nonthreshold effects in overall merger effect. To further analyze the source of heterogeneity, subgroup analysis was performed according to race, test method, sample type, and tumor stage. Subgroup analysis of tumor staging showed that the higher tumor stage according TNM stage and Duke stage had the higher sensitivity of diagnosis (Fig. 10). And in the Duke stage group, the heterogeneity was significantly reduced. The heterogeneity remained significantly in TNM stage group. Subgroup analysis of race showed that the heterogeneity was significantly decreasing in the Caucasian group, but it still existed. The heterogeneity was still significant in the Asian group. According to our data, the source of heterogeneity was not the test method and the sample type.

This meta-analysis had some limitations. Firstly, it was impossible to find out all sources of heterogeneity. The quality evaluation chart and meta-regression and subgroup analyses showed that the sources of heterogeneity may be come from paper quality of the selected articles including study size, satisfactory description of ref test, broad spectrum of disease, blinded interpretation, blinded interpretation, predesign, et al (Fig. 2). Secondly, due to language restrictions, the included studies retrieved in the article were limited to Chinese and English. Even the articles with positive results were relatively easy to publish that may be a reason leading to publication bias. Although the publication bias test showed that there was no publication bias in this study. Thirdly, some laboratories used different test reagents and cutoff values. There may be methodological bias. Fourthly, the expression of M2-PK may be related to tumor cell proliferation and metastasis. So the characteristics of tumor such as tumor pathological, tumor size, TNM stage, and degree of differentiation may be make them more heterogeneous.

## 5. Conclusion

M2-PK assay had high sensitivity and specificity for the diagnosis of CRC. Moreover, the sensitivity and specificity of the M2-PK assay were similar in stool and plasma samples. In terms of clinical significance, M2-PK in stool specimens as noninvasive screening had more clinical application prospects in early diagnosis of CRC. In the future studies, more large samples and high-quality prospective studies were needed to further confirm.

## Author contributions

**Conceptualization:** Hang Zhao, Jing Tong.

**Data curation:** Hang Zhao, Ying Wang, Jing Tong.

**Formal analysis:** Xiaonan Sun.

**Funding acquisition:** Jing Tong.

**Investigation:** Hang Zhao, Ying Wang, Jing Tong.

**Methodology:** Hang Zhao, Jing Tong.

**Project administration:** Ying Wang.

**Resources:** Hang Zhao, Xiaonan Sun, Ying Wang.

**Software:** Hang Zhao, Xiaonan Sun.

**Supervision:** Ying Wang, Jing Tong.

**Visualization:** Hang Zhao, Xiaonan Sun, Jing Tong.

**Validation:** Xiaonan Sun, Jing Tong.

**Writing—original draft:** Hang Zhao.

**Writing—review & editing:** Jing Tong.

## References

[1] Mazurek S Zwerschke W Jansen-Dürr P. Effects of the human papilloma virus HPV-16 E7 oncoprotein on glycolysis and glutaminolysis: role of pyruvate kinase type M2 and the glycolytic-enzyme complex. Biochem J. 2001;356(Pt 1):247–56. 10.1042/0264-6021:3560247 PMC1221834 11336658

[2] Lim JY Yoon SO Seol SY. Overexpression of the M2 isoform of pyruvate kinase is an adverse prognostic factor for signet ring cell gastric cancer. World J Gastroenterol. 2012;18:4037–43. 10.3748/wjg.v18.i30.4037 PMC3420001 22912555

[3] Liu Q Liang M Liu T. M2 isoform of pyruvate kinase (PKM2) is upregulated in Kazakh’s ESCC and promotes proliferation and migration of ESCC cells. Tumour Biol. 2016;37:2665–72. 10.1007/s13277-015-4073-z 26404132

[4] Lockney NA Zhang M Lu Y. Pyruvate Kinase Muscle Isoenzyme 2 (PKM2) expression is associated with overall survival in pancreatic ductal adenocarcinoma. J Gastrointest Cancer. 2015;46:390–8. 10.1007/s12029-015-9764-6 PMC7081381 26385349

[5] Papadaki C Sfakianaki M Lagoudaki E. PKM2 as a biomarker for chemosensitivity to front-line platinum-based chemotherapy in patients with metastatic non-small-cell lung cancer. Br J Cancer. 2014;111:1757–64. 10.1038/bjc.2014.492 PMC4453739 25233397

[6] Loktionov A Soubieres A Bandaletova T Mathur J Poullis A. Colorectal cancer detection by biomarker quantification in noninvasively collected colorectal mucus: preliminary comparison of 24 protein biomarkers. Eur J Gastroenterol Hepatol. 2019;31:1220–7. 10.1097/MEG.0000000000001535 31498281

[7] Yu D. Appliclibility of fecal TU M2PK detected with colloidal gold method in diagnosis of colorectal cancer. Med J Air Force. 2019;35:229–32.

[8] Dabbous HK Mohamed YAE El-Folly RF. Evaluation of fecal M2PK as a diagnostic marker in colorectal cancer. J Gastrointest Cancer. 2019;50:442–50. 10.1007/s12029-018-0088-1 29626277

[9] Ni F Xiaolin L. Value of combined detection of serum carcinoembryonic antigen，carbohydrate antigen 199 and tumor M2 pyruvate kinase for the diagnosis of colon cancer. J Xinxiang Med Univ. 2018;35:182–4.

[10] Longfeil Z Gang T Jian Y. Diagnostic of serum tumor markers for colon cancer assessed with logistic regression analysis and PLS-DA model. Chongqing Med. 2017;46:1038–41.

[11] Gang Y Lijuan T. Screening value of serum M2‐PK, CEA, CA19-9 and CA72-4 for colon cancer. Lab Med Clin. 2017;14:35–9.

[12] Rutka M Bor R Bálint A. Diagnostic accuracy of five different fecal markers for the detection of precancerous and cancerous lesions of the colorectum. Mediators Inflamm. 2016;2016:2492081. 10.1155/2016/2492081 PMC4927976 27413251

[13] Fung KY Tabor B Buckley MJ. Blood-based protein biomarker panel for the detection of colorectal cancer. PLoS One. 2015;10:e0120425. 10.1371/journal.pone.0120425 PMC4368610 25793510

[14] Sithambaram S Hilmi I Goh KL. The diagnostic accuracy of the M2 pyruvate kinase quick stool test—a rapid office based assay test for the detection of colorectal cancer. PLoS One. 2015;10:e0131616. 10.1371/journal.pone.0131616 PMC4497640 26158845

[15] Kim YC Kim JH Cheung DY. The usefulness of a novel screening kit for colorectal cancer using the immunochromatographic fecal tumor M2 pyruvate kinase test. Gut Liver. 2015;9:641–8. 10.5009/gnl13457 PMC4562782 25473070

[16] WeiJuan J. Application value of combined detection of TuM2-PK, TSGF, CEA, CA19-9 and CA242 in diagnosis of colonic cancer. Labeled Immunoassays Clin Med. 2015;22:1121–4.

[17] Wei C. Value of combined detection of tumor type M2 pyruvate kinase, tissue polypeptide specific antigen and carcino-embryonic antigen in diagnosis of colorectal cancer. Int J Lab Med. 2014;35:2775–8.

[18] Abdullah M Rani AA Simadibrata M. The value of fecal tumor M2 pyruvate kinase as a diagnostic tool for colorectal cancer screening. Acta Med Indones. 2012;44:94–9. 22745138

[19] Parente F Marino B Ilardo A. A combination of faecal tests for the detection of colon cancer: a new strategy for an appropriate selection of referrals to colonoscopy? A prospective multicentre Italian study. Eur J Gastroenterol Hepatol. 2012;24:1145–52. 10.1097/MEG.0b013e328355cc79 22735608

[20] Meng W Zhu H-H Xu Z-F. Serum M2-pyruvate kinase: a promising non-invasive biomarker for colorectal cancer mass screening. World J Gastrointest Oncol. 2012;4:145–51. 10.4251/wjgo.v4.i6.145 PMC3382661 22737276

[21] Yu Z Lei W Jizhong G. The value of tumor M2 pyruvate kinase in diagnosis of colon cancer. Jiangsu Med J. 2011;37:523–5.

[22] Yong L Junjiang W. Clinical significance of blood and fecal tumor M2-pyruvate kinase expression in patients with colorectal cancer. J South Med Univ. 2011;31:2087–9. 22200720

[23] Shastri YM Loitsch S Hoepffner N. Comparison of an established simple office-based immunological FOBT with fecal tumor pyruvate kinase type M2 (M2-PK) for colorectal cancer screening: prospective multicenter study. Am J Gastroenterol. 2008;103:1496–504. 10.1111/j.1572-0241.2008.01824.x 18510609

[24] Lianzhou C. Expression and clinical significance of tumor M2 pyruvate kinase in colorectal cancer patients. Chin J Health Lab Technol. 2008;18:1830–1.

[25] Haug U Rothenbacher D Wente MN Seiler CM Stegmaier C Brenner H. Tumour M2-PK as a stool marker for colorectal cancer: comparative analysis in a large sample of unselected older adults vs colorectal cancer patients. Br J Cancer. 2007;96:1329–34. 10.1038/sj.bjc.6603712 PMC2360192 17406361

[26] Koss K Maxton D Jankowski JA. Faecal dimeric M2 pyruvate kinase in colorectal cancer and polyps correlates with tumour staging and surgical intervention. Colorectal Dis. 2008;10:244–8. 10.1111/j.1463-1318.2007.01334.x 17784868

[27] Mulder SA van Leerdam ME van Vuuren AJ. Tumor pyruvate kinase isoenzyme type M2 and immunochemical fecal occult blood test: performance in screening for colorectal cancer. Eur J Gastroenterol Hepatol. 2007;19:878–82. 10.1097/MEG.0b013e3282cfa49c 17873612

[28] Zhang B Chen J-Y Wang G-B. Value of fecal tumor M2 pyruvate kinase in diadnosis of colorectal cancer. World Chin J Digestol. 2007;15:193–6.

[29] Guan-Fu WJ Yi-Hua LP Xin-Min S. Evaluation of fecal tumor M2 pyruvate kinase combined with fecal occult blood test as a detecting tool for colorectal cancer. J Sun Yat-sen Univ. 2006;27:350–3.

[30] Tonus C Neupert G Sellinger M. Colorectal cancer screening by non-invasive metabolic biomarker fecal tumor M2-PK. World J Gastroenterol. 2006;12:7007–11. 10.3748/wjg.v12.i43.7007 PMC4087345 17109496

[31] Hardt PD Mazurek S Toepler M. Faecal tumour M2 pyruvate kinase: a new, sensitive screening tool for colorectal cancer. Br J Cancer. 2004;91:980–4. 10.1038/sj.bjc.6602033 PMC2409989 15266315

[32] Naumann M Schaum B Oremek GM. Faecal pyruvate kinase type M2—a valid screening parameter for colorectal cancer? Preliminary results from a multicenter comparative study. Dtsch Med Wochenschr. 2004;129:1806–7. 10.1055/s-2004-829033 15314744

[33] Chen Y Gao S-G Chen J-M. Serum CA242, CA199, CA125, CEA, and TSGF are biomarkers for the efficacy and prognosis of cryoablation in pancreatic cancer patients. Cell Biochem Biophys. 2015;71:1287–91. 10.1007/s12013-014-0345-2 25486903

[34] Fatela-Cantillo D Fernandez-Suarez A Moreno MAM. Prognostic value of plasmatic tumor M2 pyruvate kinase and carcinoembryonic antigen in the survival of colorectal cancer patients. Tumour Biol. 2012;33:825–32. 10.1007/s13277-011-0304-0 22231432

[35] Palsson-McDermott EM Curtis AM Goel G. Pyruvate kinase M2 regulates Hif-1α activity and IL-1β induction and is a critical determinant of the warburg effect in LPS-activated macrophages. Cell Metab. 2015;21:65–80. 10.1016/j.cmet.2014.12.005 PMC5198835 25565206

[36] Nicholson BD Shinkins B Pathiraja I. Blood CEA levels for detecting recurrent colorectal cancer. Cochrane Database Syst Rev. 1134;2015:Cd01. 10.1002/14651858.CD011134.pub2 PMC7092609 26661580

[37] Fan P Zhang Y Deng F. The value of serum human tumor protein P53 in colorectal cancer combined diagnosis and postoperative monitoring [in Chinese]. Zhonghua Yi Xue Za Zhi 2017;97:2670–3. 10.3760/cma.j.issn.0376-2491.2017.34.006 28910954

[38] Hardt PD Toepler M Ngoumou B. Measurement of fecal pyruvate kinase type M2 (tumor M2-PK) concentrations in patients with gastric cancer, colorectal cancer, colorectal adenomas and controls. Anticancer Res. 2003;23:851–3. 12820312

[39] Muñoz-Colmenero A Fernández-Suárez A Fatela-Cantillo D. Plasma tumor M2-Pyruvate kinase levels in different cancer types. Anticancer Res. 2015;35:4271–6. 26124389

[40] Higgins JP Thompson SG. Quantifying heterogeneity in a meta-analysis. Stat Med. 2002;21:1539–58. 10.1002/sim.1186 12111919

